# Transcriptomic genotyping elucidates the population structure and demographic history of the endangered poison frog *Oophaga vicentei* (Anura: Dendrobatidae)

**DOI:** 10.1186/s12864-026-12957-8

**Published:** 2026-06-09

**Authors:** Anaisa Cajigas Gandia, Heike Pröhl, Vasiliki Mantzana Oikonomaki, Roberto Ibáñez, Ariel Rodríguez

**Affiliations:** 1 Stiftung Tierärztliche Hochschule Hannover, Institut für Zoologie, Bünteweg 17, Hannover, 30559 Germany; 2 Smithsonian Tropical Research Institute, Panama, Apartado Postal 0843-03092 Panama; 3 Sistema Nacional de Investigación, Secretaría Nacional de Ciencia, Tecnología e Innovación, Panama, Apartado Postal 0816-02852 Panama

**Keywords:** Demography, Diversity, Endangered, Frogs, *Oophaga*, Population genetics, Structure

## Abstract

**Background:**

Amphibians are among the most vulnerable vertebrates, with 41% of assessed species at risk of extinction. The genus *Oophaga* has most of its species currently listed as threatened by the IUCN. *Oophaga vicentei*, an endangered species endemic to central Panama, is facing strong pressures from habitat fragmentation and mining. Despite its threat status, little is known about its population structure and size. Reduced representation approaches are cost efficient alternative to obtain genome-wide estimates of genetic diversity in non-model species. We herein genotyped thousands of exonic SNPs using RNA-seq data to uncover the genetic structure and effective population size of *O. vicentei*.

**Results:**

Population structure analyses revealed two main genetic clusters: a West group (formed by individuals of Calovébora, La Empalizada, and Loma Grande) and an East group with individuals from La Ceiba. A further subdivision of the West cluster was apparent on the PCA with two localities (Calobévora, La Empalizada) splitting from the other Loma Grande. The La Ceiba locality was genetically the most divergent from the rest, with Calovébora and La Empalizada being the most similar. Effective population size of *O. vicentei* peaked around the Last Interglacial period and declined after the Last Glacial Maximum, with the locality La Ceiba showing the most pronounced decline towards present time.

**Conclusions:**

The significant amount of data we obtained validates the applicability of mRNA sequences to elucidate the population structure and demography of a species. Our results reveal strong genetic structure in *O. vicentei*, with the two main population clusters showing significant differences in their most recent demographic history. These findings provide a genomic baseline for conservation strategies, emphasizing the need to protect all known populations of this endangered species.

**Supplementary Information:**

The online version contains supplementary material available at 10.1186/s12864-026-12957-8.

## Background

Amphibians are among the most threatened vertebrate taxa, with 41% of assessed species estimated to be declining over the past 60 years [1–3]. They play essential ecological roles in maintaining ecosystem health, but their permeable skin and biphasic life cycles make them highly sensitive to environmental disturbance. Multiple threats to amphibian´s survival have been identified in the last 40 years across the world, including habitat loss and fragmentation, climate change, emerging infectious diseases, and overexploitation [1, 4].

Particularly in the Neotropics, amphibians represent one of the richest and most endangered vertebrate groups [5]. Across this region, poison frogs of the genus *Oophaga* (Dendrobatidae) are of conservation concern. Eleven of the 12 currently recognized species are threatened according to the IUCN [3], and four are considered Evolutionarily Distinct and Globally Endangered (EDGE) species (https://www.edgeofexistence.org). Population genetic studies of closely related species within this genus have revealed strong population differentiation at fine spatial scales, reflecting the combined effects of limited dispersal, geographic barriers, and ecological specialization [6–9]. These findings highlight the importance of examining genetic variation in other narrowly distributed *Oophaga* species, where local adaptation and reduced connectivity may have significant implications for conservation.

Vicente’s poison frog (*Oophaga vicentei*) is a small (< 24 mm) dendrobatid endemic to Veraguas, Colón and Coclé provinces, in central Panama [10]. It inhabits bromeliads in the arboreal stratum [11], as well as leaf litter and rocks on the forest floor, which suggests a broader microhabitat use and potential ecological plasticity [12]. It exhibits remarkable warning color diversity, including yellow, red, blue, and green morphs with variable black reticulations or spotted patterns [13]. These striking phenotypes make the species highly attractive for the illegal pet trade, which together with chytridiomycosis [14], habitat loss, and fragmentation, constitutes the main threats to the species, whose current population size is unknown [10]. Consequently, *O. vicentei* is listed as Endangered in the IUCN Red List [10] and included in CITES Appendix II. These multiple pressures raise serious concerns about the long-term viability of the species, as small and fragmented populations are at risk of losing rare alleles, increasing homozygosity, and suffering reduced adaptive potential [15, 16].

In recent years, with the rapid development of next-generation sequencing techniques, conservation research has advanced into a new era of molecular ecology. This has provided unprecedented access to genomic data that deepens our understanding of genetic diversity, population structure, and adaptive variation in endangered species. Particularly, the use of Single Nucleotide Polymorphism (SNP) markers offers powerful tools to assess population genetics and demographic dynamics, as well as extinction risk [17–19], thereby enabling more informed decisions in species management, breeding, and restoration programs for endangered species [20]. Overall, amphibians lag far behind other vertebrates in the number of sequenced genomes. For most species, the messenger RNA sequencing (RNA-seq) of the expressed fraction of the genome represents a cost-effective alternative that is increasingly being used in conservation genetics of this threatened vertebrate group [21].

Here, we use existing RNA-seq data to characterize the genetic diversity of *O. vicentei*. Specifically, we investigate levels of genetic variation among individuals of different localities, evaluate patterns of genetic divergence, and infer ecological and historical processes shaping population structure. Our findings can provide critical baseline data for conservation planning while clarifying important aspects of the evolutionary history of this poison frog.

## Methods

### Data collection

**Fig. 1:**
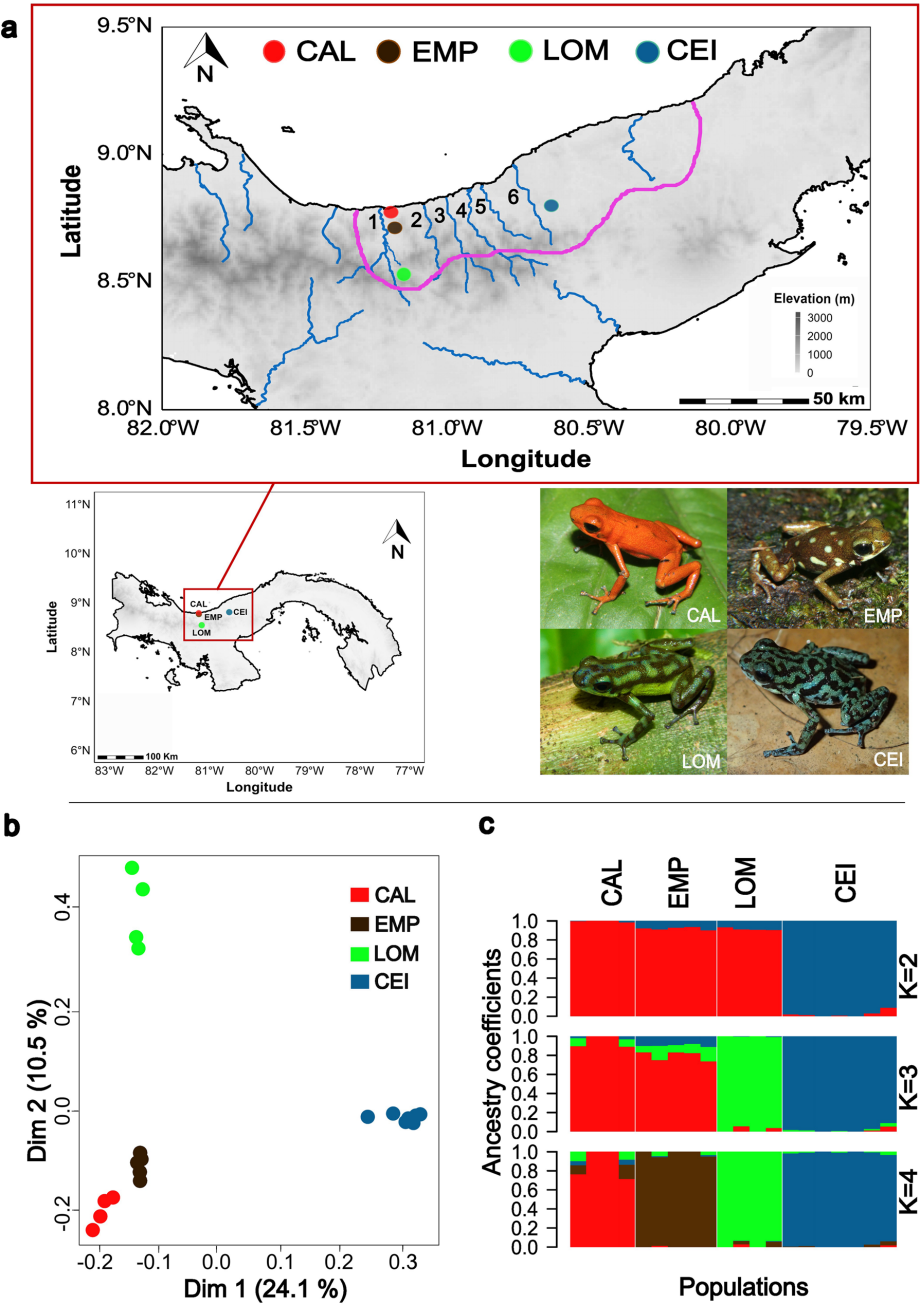
Population genetic structure of *Oophaga vicentei*. **A** Geographic distribution and color morphs of sampled localities in Panama. The pink polygon represents the current known distribution area of the species according to the IUCN. Numbers stand for: 1 Calobévora river, 2 Guázaro river, 3 Concepción river, 4 Veraguas river, 5 Belén river, 6 Petaquilla river. **B** Principal Component Analysis. **C** Admixture analysis. Photos in A) were taken by Ariel Rodríguez (CAL, EMP, LOM) and Roberto Ibáñez (CEI)

We used available RNA-seq data from skin, liver and eyes of *O. vicentei* (NCBI SRA Bioproject PRJNA1234489, with accession numbers: SAMN47383778 – SAMN47383821). These samples cover four different color morphs across four localities (Fig. 1A): Calovébora (CAL: 8.789° N, 81.215° W; elevation: 8 m; *N* = 4; color morph: red), La Empalizada (EMP: 8.761° N, 81.214° W; elevation: 82 m; *N* = 5; color morph: brown dotted), La Ceiba (CEI: 8.808° N, 80.607° W; elevation: 99 m; *N* = 7; color morph: aquamarine) and Loma Grande (LOM: 8.542° N, 81.158° W; elevation: 782 m; *N* = 4; color morph: green). Additional details on sample collection, sequencing, the phenotypes and associated metadata can be found in [22].

### Reference genome annotation

The genome assembly of *Oophaga sylvatica* (NCBI accession: GCA_033576555.1) was used as reference for this study. This is the most complete genome assembly of the genus *Oophaga* to date, but it lacks annotation. To develop an annotation for this genome, we started by identifying chromosome-level scaffolds. Relative chromosome sizes were estimated from a karyotype image of an individual *O. sylvatica* from Quingüe, Ecuador [23]. We used ImageJ [24] area measurements of each pair of chromosomes in a binary (black and white) version of the karyotype image and estimated the relative chromatin size of each chromosome as its image area divided by the total area occupied by chromosomes in the image. Sorted values of scaffold sizes (in MB) were regressed against sorted chromatin sizes to estimate scaffolding consistency. Genome assembly stats, contiguity and completeness metrics [25] were estimated with BlobToolKit [26].

We identified repetitive elements using RepeatModeler2 [27] fitted with Dfam v3.7 [28] and RepBase v20181026 [29] databases. We used the repeats classification file resulting from RepeatModeler2 as input for RepeatMasker v4.1.2 [30] and then used the repeat annotation output to soft mask repeats sequences in the genome using *maskfasta* option in BEDTools v2.26 [31]. We then annotated the soft-masked version of the *O. sylvatica* genome scaffolds using GALBA [32], a suite of algorithms that uses high confidence alignments of protein sequences to the genome to train the gene prediction tool AUGUSTUS [33]. The proteome of *O. sylvatica* obtained in a previous study [34] was used as input for GALBA, after removing very small sequences (< 100 aa).

### Genotype calling

We performed adapter removal and quality trimming of RNA reads with FastP v0.23.4 [35]. Then, we aligned all RNA-seq reads using the two-pass mode of STAR and default settings [36]. After, we used the GATK pipeline [37] following the best practices workflow and recommendations, for RNA-seq based haplotype calling [38]. The pipeline included the following steps (GATK tools in parenthesis): identifying duplicate reads (MarkDuplicates); merging tissue reads per individual (samtools merge); splitting exon spanning reads and clip sequences overhanging the intronic regions (SplitNCigarReads); variant calling per individual (HaplotypeCaller); joint variant calling of all individuals (CombineGVCFs); and quality filtering (VariantFiltration) to include only bi-allelic SNPs with Fisher strand values (FS) > 30.0 and quality by depth values (QD) < 2.0. The resulting genotype file was filtered further using VCFtools v0.1.16 [39] and the *O. sylvatica* annotation to only keep SNPs outside coding sequences to obtain putatively neutral SNPs with minimum depth (minDP) ≥ 10, minor allele count (mac) ≥ 3, and < 25% of missing data (max-missing) ≥ 0.75. Remaining SNPs were then pruned for linkage disequilibrium (LD) using the “--indep-pairwise” option in PLINK v2.0 [40] with the following configuration (window size: 50, variant count: 1, squared correlation threshold: 0.7). A final dataset of putatively 26 296 neutral and unlinked SNPs was retained for all population genetic analyses.

### Population genetic analyses

Population genetic analyses were performed using the *sambaR* package [41] in R v4.4.1 [42].

Genetic structure was examined through multivariate ordination methods, including Principal Component Analysis (PCA) [43], Discriminant Analysis of Principal Components (DAPC) [44] and Principal Coordinates Analysis (PCoA) [45]. These complementary approaches allowed us to investigate clustering patterns and relationships among localities, as well as to evaluate the consistency of genetic differentiation across both unsupervised (PCA, PCoA) and supervised (DAPC) frameworks. In addition, admixture levels were inferred with LEA (*nruns* = 50, *kmin* = 1, *kmax* = 4). The maximum number of clusters (*K* = 4) was set to match the number of sampling localities, and the most likely *K* value was estimated with the cross-entropy criterion [46]. We assessed genetic diversity by analyzing nucleotide diversity (π), the population mutation rate based on the Watterson estimator theta (Θ) [47], minor allele frequencies, and multi-locus heterozygosity. To quantify population genetic divergence, we calculated Nei´s distance [48], and Weir & Cockerham 1984 *Fst* [49]. Values of Nei’s genetic distance greater than 0.15 and *Fst* values exceeding 0.25 were considered indicative of high genetic differentiation [50].

### Population demographic history

We estimated the genotype likelihoods per site (*doSaf − 1*) for each of the ancestral populations separately with ANGSD v0.938 [51]. We used the GATK genome-based read mappings as input and the following SNP filters: minimum – maximum depth: 1-50x, keep sites covered on all individuals per pop with minimum mapping quality: 20, discard wrongly mapped reads, reads that do not map uniquely, and keep only paired-end reads that mapped correctly (only_proper_pairs). We also filtered out protein-coding sites, using –sites and –rf options, to exclude potential confounding effects of sites under selection. Because population structure and sample size have proven to greatly affect demographic inferences [52–58], we here used the realSFS tool of ANGSD to estimate the folded site frequency spectrum (SFS, distribution of allele frequencies across all sites) for the two main clusters identified by LEA analysis while keeping an adequate sample size for demographic estimations [57]. The folded SFS estimates were used as input for Stairway Plot v2.1 [59] to infer the demographic history of the West and East clusters using 200 bootstrap replicates of the SFS and assuming a mutation rate of 1e − 9 mutations per site per year [60–62] and a generation time of one year [63].

## Results

### Genome annotation

The assembled genome of *O. sylvatica* contains 15 283 scaffolds. The first 10 scaffolds contain 85% of the data and are distinctly larger than the other scaffolds (Fig. S1). This number matches the published karyotype (*N* = 10 [23]) for that species, and the scaffold sizes are strongly correlated with chromosome sizes estimated from the karyotype (Fig. S1). These first 10 scaffolds are likely to represent chromosome-level assemblies.

Repeat modelling analyses identified 81% of the *O. sylvatica* genome as repetitive elements, and most of the identified sequences represented interspersed repeats. Most of these classified repeats were retroelements (40.6% of the genome), with the Gypsy/DIRS1 family of Long Terminal Repeats (LTRs) being the most abundant (27% of the genome). DNA transposons covered another 18.3% of the genome, with Tc1-IS630-Pogo and hobo-Activator sequences being the most abundant (7.5% and 5.1%, respectively). A total of 22% of the repetitive sequences in the genome could not be classified. Summary statistics of the RepeatModeler2 pipeline results are presented in Table S1. After soft-masking repetitive regions, our genome annotation identified 55 565 gene models in the assembly. The annotation included an average of 1.2 transcripts per gene and 3.8 exons per coding sequence (CDS). The average gene length is 20 830 bp, with a mean CDS length of 1022 bp.

### Population genetics

Transcriptome-based variant calling yielded 7 812 553 initial SNPs across all individuals. After quality and biallelic filtering, 3 856 452 SNPs remained. Subsequent filtering to keep variants in non-coding regions based on depth, minor allele count, missing data thresholds, and linkage disequilibrium produced a final set of 26 296 putatively neutral and unlinked SNPs, that were retained for downstream population genetic analyses.

Population structure analyses yielded consistent patterns across multivariate approaches (PCA, PCoA and DAPC). The PCA revealed two main genetic clusters: a West cluster comprising the Calovébora (CAL), La Empalizada (EMP), and Loma Grande (LOM) localities, and an East cluster represented by the La Ceiba (CEI) (Fig. 1B). Within the West cluster, a further subdivision into two subclusters was evident, one including the red individuals from Calovébora and the brown-dotted individuals from La Empalizada, and another corresponding to the green individuals from Loma Grande. The same clustering was observed in PCoA and DAPC results (Fig. S2). Admixture analyses also indicated that the most likely number of ancestral populations is two (Fig. 1C, Fig. S2).

The genetic diversity in Calovébora and La Empalizada is slightly higher than in the other two localities (Table 1, Fig. S3). Pairwise *Fst* ranged from 0.130 to 0.350, with Calovébora and La Empalizada being more similar, while La Ceiba is the most divergent locality. The same pattern is shown by the Nei´s genetic distance, which ranges from 0.104 to 0.223 (Table 2).

**Table 1:** Population genetic diversity of sampled localities of *Oophaga vicentei *. Minor allele frequency (MAF), scaled Multi-locus heterozygosity (MHLs), scaled Nucleotide diversity (π), and scaled Watterson estimator theta (Θs)

Population	Metapopulation	CAL	EMP	LOM	CEI
MAF	0.179	0.194	0.198	0.179	0.159
MLHs	0.999	1.190	1.048	0.976	0.868
π	0.003288	0.002995	0.002941	0.002714	0.002320
Θs	0.2698	0.2692	0.2917	0.2775	0.2270

**Table 2:** Population genetics divergence among sampled localities of *Oophaga vicentei *. Pairwise Weir & Cockerham 1984 fixation index (*Fst*) values are shown above the diagonal, and Nei’s genetic distances are shown below the diagonal. The highest values per pairwise comparison are highlighted in bold and indicate strong genetic differentiation

	CAL	EMP	LOM	CEI
CAL		0.130	0.182	0.350
EMP	0.104		0.155	**0.302**
LOM	0.130	0.122		**0.339**
CEI	**0.223**	**0.192**	**0.213**	

### Population demographic history

**Fig. 2:**
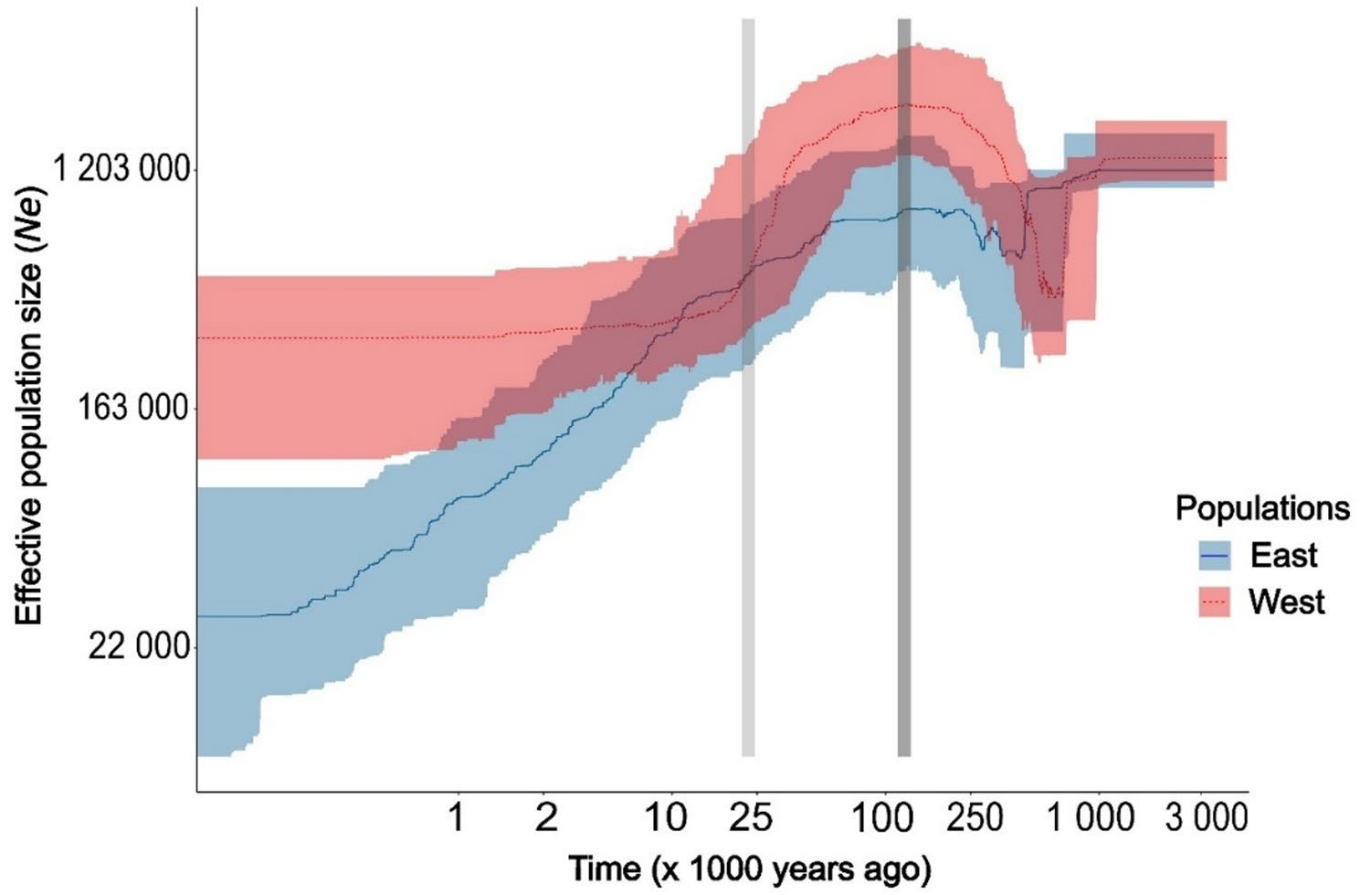
Demographic trends for the two main genetic clusters of *Oophaga vicentei* including the West (red trace) and the East (blue trace) population clusters. The line traces represent the median estimates of population size and the shaded areas their 75% confidence intervals. The gray vertical bands indicate the Last Interglacial (warm climate, dark gray) and the Last Glacial Maximum (cold climate, light gray) periods. Both axes are in logarithmic scale

Based on site frequency spectrum analysis, we estimate that the effective population size of *O. vicentei* peaked around the Last Interglacial (LIG) climatic period (~ 125 000 years ago) to a median of 870 thousand individuals for the East cluster and 2.0 million for the West cluster. After this period, the effective population size decreased in both ancestral populations until the Last Glacial Maximum (LGM) (~ 25 000 years ago). From this point onwards the populations of the West cluster stabilized on a median of 294.5 thousand individuals at present time, while the East cluster continued its decrease reaching an effective population size of 28.7 thousand individuals at present (Fig. 2).

## Discussion

Understanding the genetic structure and demographic history of endangered amphibians is essential for assessing their evolutionary potential and informing conservation actions. In this context, population genetics research of non-model organisms has benefitted from the use of single nucleotide polymorphisms given their abundance across the genome and their power to resolve patterns of genetic diversity, structure and demographic history, even with relatively modest sample sizes [64, 65]. In this study, we demonstrate the utility of transcriptome-based approaches for population genomic inference in an endangered amphibian, successfully generating thousands of informative SNPs and site frequency spectra from Illumina mRNA sequencing data. Our findings reveal that *O. vicentei* exhibits a pronounced genetic structuring with the samples forming two main clusters that show important differences in genetic diversity and demography, likely shaped by past climatic events.

### Population genetics

Forest-specialist anuran species occurring in regions of high topographic complexity tend to show a strong phylogeographic structure [66]. Sampled localities of *O. vicentei* formed two main genetic clusters, one comprising the three western sites and a second corresponding to CEI, across a topographically complex area of the Caribbean versant of the Central Mountains of Panama. This pattern indicates restricted gene flow and strong genetic differentiation at a relatively fine spatial scale. Similar levels of structuring have been reported in other dendrobatids, including *Oophaga pumilio* and *Oophaga granulifera*, where geographic barriers, limited dispersal, and color-based assortative mating restrict gene flow [7, 67]. Despite multiple studies have shown that it is possible to obtain reliable population structure, divergence and demography estimates with relatively small sample sizes [65, 68–71], the observed structure in this study could be biased towards the geographic distribution of sampled localities, and therefore its specific historical and ecological drivers cannot be resolved with the present dataset. However, landscape features such as complex topography, including river systems, have been implicated in shaping amphibian population differentiation in other regions [72–75], and may represent plausible contributing factors in this case. Additional studies including more localities within the distribution area and integrating landscape, ecological, and genomic data will be required to explicitly test these hypotheses in *O. vicentei*.

Genetic diversity forms the basis of all biological variation, underpinning the ability of a species to adapt to changing environmental conditions. When this diversity is reduced, it can diminish evolutionary potential and elevate the risk of extinction for species and populations [76, 77]. We estimated the nucleotide diversity in *O. vicentei* as π ≈ 0.0033. To our knowledge, the only available diversity estimates in the genus *Oophaga* come from *O. sylvatica* (π = 0.0056) and *O. pumilio* (π = 0.0069), which were derived from mitochondrial DNA and microsatellite markers, respectively [7, 55]. Because these marker systems typically yield higher estimates than genome-wide SNP data, direct quantitative comparisons are inappropriate. Although the number of genomic studies in *Oophaga* has increased in recent years, genome-wide estimates of population genetic diversity remain scarce, highlighting the need for more comparable datasets to place diversity levels of endangered species such as *O. vicentei* into a broader evolutionary and conservation context.

### High phenotypic diversity despite close geographic proximity

High genetic differentiation between the West and East clusters supports restricted gene flow, limited dispersal and connectivity, potentially shaped by geographic and environmental heterogeneity. Within the western cluster, sampled localities also exhibit genetic differentiation, although divergence among them is lower than that observed between the West and East clusters. This supports the phenotypic diversity observed, with each locality displaying a distinct color and pattern (Fig. 1A). Particularly interesting is the case of CAL and EMP, that show radically distinct phenotypes despite their proximity (e.g. less than 3 linear km distance) while showing the lowest genetic divergence of all pairwise comparisons.

In dendrobatids, coloration functions both as a cue in mate choice [78–80], and as warning signal to predators [81–83], allowing sexual and natural selection to act in concert. Sexual imprinting (i.e. offspring learn parental phenotypes as the basis of their own mate preferences [84]), may reinforce local color preferences and sustain distinct phenotypes even over short geographic distances. This mechanism has been reported in *O. pumilio*, where cross-fostered females prefer to court mates of the same color as their foster mother, and cross-fostered males are more aggressive towards rivals that share the color of their foster mother [85]. Strong sexual selection on color morphs may maintain distinct phenotypes through assortative mating, which can prevent the homogenization of visual traits, as occurs in *O. pumilio* [67, 79, 86]. However, given the close distance between these three localities, and the absence of clear geographic barriers between them, we believe that a combination of strong philopatry [87], small home ranges [88, 89] and sexual imprinting [85] may explain this extreme phenotypic diversification, although further sampling will be required to test these hypotheses explicitly.

### Demographic history

Demographic inferences suggest that past climatic oscillations during the Late Pleistocene likely shaped the diversity and effective population sizes of *O. vicentei*. The effective population size trajectory indicates that this species experienced a demographic peak around the LIG, followed by a gradual decline after the LGM. However, given our small sample size these results should be considered as a first approximation, and more sampling would be desirable in the future to precisely estimate the demographic history of this species. Nevertheless, our data is consistent with that of other Neotropical amphibians that experienced climate-driven range shifts and subsequent isolation in forest refugia [90, 91].

While the West cluster shows signs of demographic recovery following the population reduction that occurred after the LGM, the East cluster (CEI locality) does not show comparable evidence of recovery and may still be experiencing reduced effective population size. However, the drivers underlying these contrasting demographic trajectories remain uncertain. Possible explanations include differential responses to post-glacial environmental changes, and local ecological conditions. Additional ecological, genomic, and environmental data will be required to disentangle the relative contributions of these factors.

### Contemporary pressures and anthropogenic impacts

While historical processes shaped much of *O. vicentei* genetic landscape, recent anthropogenic pressures, such as habitat loss driven by deforestation and mining [92] are threatening this species with extinction. These factors have recently exacerbated in areas adjacent to La Ceiba, where large-scale mining operations along with ongoing deforestation activities, have caused extensive habitat degradation within the Donoso Protected Area [93]. The actual impacts of these activities on the genetically unique and declining population of *O. vicentei* are of high concern given our results. Moreover, the CAL and EMP localities occur outside protected areas, where deforestation and habitat degradation are also frequent. Even within Santa Fe National Park, where the LOM locality occurs, habitat disturbances are evident, although seemingly less severe than in the Donoso region.

### Conservation implications

From a conservation standpoint, our results emphasize the importance of incorporating genetic structure into management planning for *O. vicentei*. The two major genetic clusters identified across sampled localities represent distinct components of the species’ evolutionary diversity and should be considered when defining conservation and management priorities. Although overall genetic diversity is relatively low and demographic reconstructions suggest population declines, these inferences must be interpreted cautiously given the limited sample sizes and the restricted number of sampling localities, which reflect both logistical constraints and the apparent rarity of the species in the field. Nonetheless, the observed genetic structure provides valuable insights into spatial patterns of differentiation.

The recent rediscovery of a population in Cerro Gaital and the identification of an additional population further east near Bajo Bonito confirm the presence of the species in the El Valle region where it was considered extinct [10]. Both populations are phenotypically similar to the aquamarine morph of the eastern cluster [94], suggesting that this lineage may have a broader distribution than previously recognized. These newly identified populations should be incorporated into future genetic and ecological assessments, as they may refine the delineation of conservation units and improve our understanding of connectivity across the species’ range. Overall, these findings highlight the importance of preserving habitat connectivity and considering multiple genetic lineages in potential *ex situ* breeding or reinforcement programs. Such measures may help maintain genetic and phenotypic diversity in the face of ongoing threats from habitat loss, disease, climate change, and illegal trade.

## Conclusions

This study provides the first population genetic characterization of the endangered poison frog *Oophaga vicentei*, revealing pronounced genetic structuring and historical demographic shifts across its range in central Panama. Two main distinct genetic clusters were identified, separating the western localities of Calovébora, La Empalizada, and Loma Grande from the eastern locality La Ceiba. These patterns indicate limited gene flow and strong isolation among sampled localities. Historical demographic reconstructions revealed population expansions during the LIG period, followed by steady declines after the LGM, consistent with climate-driven habitat changes. Together, these findings highlight the combined influence of geography and historical processes on the genetic landscape of *O. vicentei* and underscore the importance of considering fine-scale genetic structure in conservation planning. Future research should integrate genomic, ecological, and behavioral data of additional localities to better delineate the population structure and forces responsible for generating and maintaining the intriguing phenotype diversity of this species. Conservation efforts should focus on protecting all known populations, promoting habitat connectivity, and monitoring genetic diversity over time. By establishing a genomic diversity baseline for *O. vicentei*, this study should contribute to both the preservation of this endangered amphibian and the broader understanding of diversification and resilience in Neotropical poison frogs.

## Supplementary Information

Supplementary Material 1. MS Word document (docx) with the supplementary figures (Figure S1-S3) and Table S1, showing the summary statistics for the annotation of *Oophaga sylvatica* genome, and additional population genetic structure and diversity analyses of *Oophaga vicentei*.

## Data Availability

The raw RNA reads used in this study are available at NCBI SRA Bioproject PRJNA1234489, with accession numbers: SAMN47383778 – SAMN47383821. The *O. sylvatica* reference genome annotation, as well as the VCF and PLINK files containing all the 26 296 SNPs used in the analyses are available on Zenodo (10.5281/zenodo.19609831). Scripts used for data pre-processing, haplotype calling, SNP filtering, and downstream population genetic analyses are available on GitHub ( https://github.com/cajigas/vicentei_population_genetics).

## Abbreviations

aa: Amino-acid
ANGSD: Analysis of Next Generation Sequencing Data
bp: Base pairs
BUSCO: Benchmarking Universal Single-Copy Orthologs
CAL: Calovébora
CEI: La Ceiba
CDS: Coding Sequences
DNA: Deoxyribonucleic Acid
EDGE: Evolutionarily Distinct and Globally Endangered
EMP: La Empalizada
*Fst*: Fixation index
GALBA: Genome Annotation with Large-scale Biologically Aligned protein evidence
GATK: Genome Analysis Toolkit
IUCN: International Union for Conservation of Nature
LEA: Landscape and Ecological Association
LD: Linkage Disequilibrium
LGM: Last Glacial Maximum
LIG: Last Interglacial
LOM: Loma Grande
LTRs: Long Terminal Repeats
MAF: Minor Allele Frequency
MB: Megabytes
MHLs: Multi-locus Heterozygosity
NCBI: National Center for Biotechnology Information
*Ne*: Effective Population Size
PCA: Principal Components Analysis
RNA-seq: Ribonucleic acid (RNA) sequencing
SRA: Sequence Read Archive
STAR: Spliced Transcripts Alignment to a Reference
SNPs: Single Nucleotide Polymorphisms
