# Supplementary material for: Transcriptomic genotyping elucidates the population structure and demographic history of the endangered poison frog Oophaga vicentei (Anura: Dendrobatidae)
Source: BMC Genomics. 2026 Jun 9;27:554. doi: 10.1186/s12864-026-12957-8 (PMC13281361; doi:10.1186/s12864-026-12957-8)
Supplement: Supplementary file 1 — Supplementary Material 1. MS Word document (docx) with the supplementary figures (Figure S1-S3) and Table S1, showing the summary statistics for the annotation of Oophaga sylvatica genome, and additional population genetic structure and diversity analyses of Oophaga vicentei. [file 12864_2026_12957_MOESM1_ESM.docx]

**SUPPLEMENTARY MATERIAL**

to be published in support of the article:

**Transcriptomic genotyping elucidates the population structure and demographic history of the endangered poison frog *Oophaga vicentei*** **(Anura: Dendrobatidae)**

Anaisa Cajigas Gandia, Heike Pröhl, Vasiliki Mantzana Oikonomaki, Roberto Ibáñez, Ariel Rodríguez

**Important note:** Figures and tables are provided in the order they appear cited in the main manuscript, to make it more understandable and easier to follow.

**Figure S1.** Scaffold statistics, completeness and consistency of the *Oophaga sylvatica* genome assembly.

**Table S1.** Summary statistics of the repeat scan and identification in *Oophaga sylvatica* genome.

**Figure S2.** Population genetic structure of *Oophaga vicentei*.

**Figure S3.** Population genetic diversity of *Oophaga vicentei.*


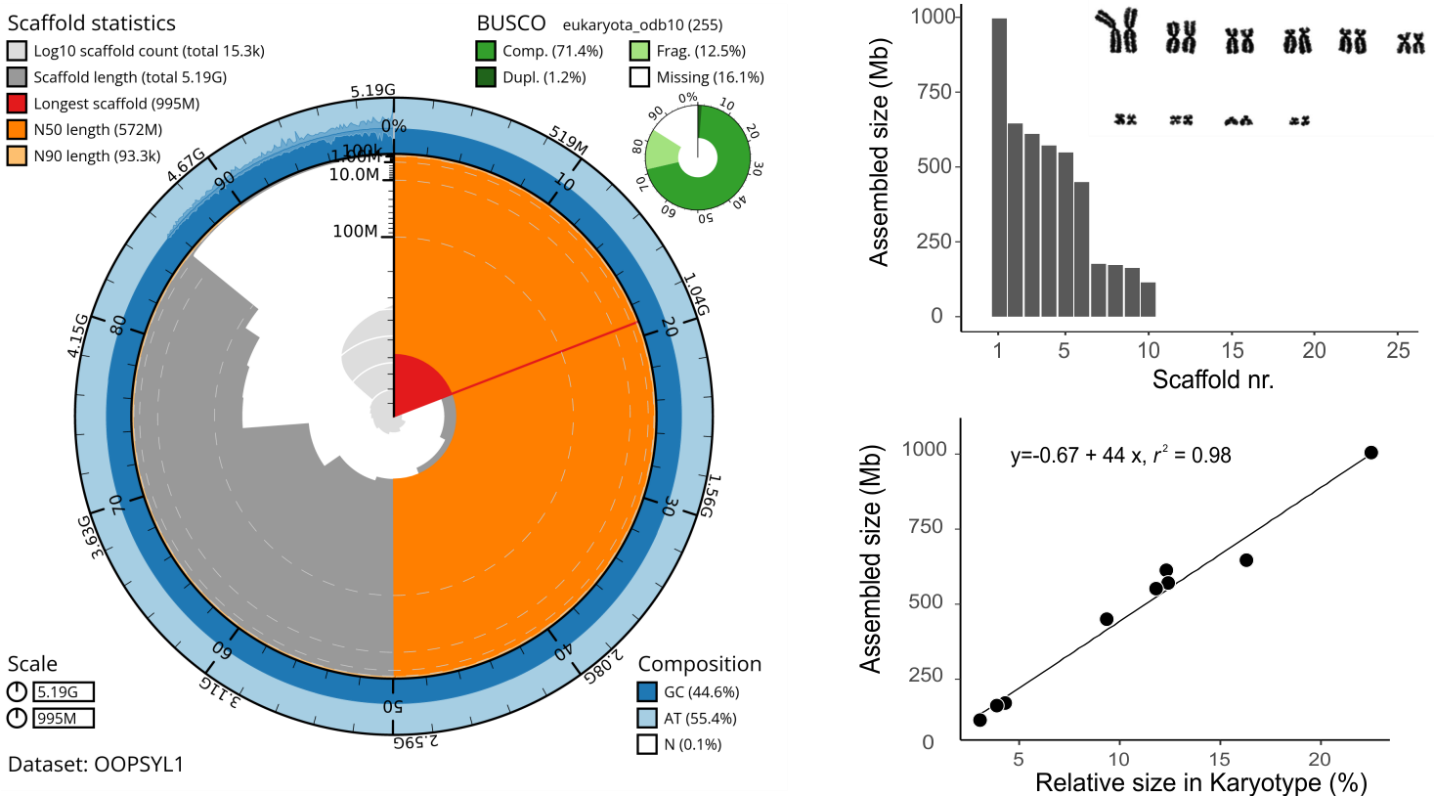


**a**

**b**

**c**

**Figure S1.** Scaffold statistics, completeness and consistency of the *Oophaga sylvatica* genome assembly. A) Snail plot showing the scaffolding statistics and BUSCO scores of the assembly. B) Bar plot of scaffold lengths showing the much larger size of the first 10 scaffold, the inset shows the karyotype of an individual *O. sylvatica* (n = 10) from Quingüe population (1). C) Relationship between scaffolded length of the first 10 scaffolds and relative chromosome sizes, as estimated from the karyotype.

**Table S1.** Summary statistics of the repeat scan and identification in *Oophaga sylvatica* genome.

|  | **number of elements*** | **length (Mb)** | **percentage (%)** |
| --- | --- | --- | --- |
| *Retroelements* | 1662784 | 2108.31 | 40.65 |
| SINEs: | 19004 | 1.88 | 0.04 |
| Penelope | 331259 | 328.57 | 6.33 |
| LINEs: | 686786 | 612.60 | 11.81 |
| CRE/SLACS | 0 | 0 | 0 |
| L2/CR1/Rex | 269378 | 221.95 | 4.28 |
| R1/LOA/Jockey | 0 | 0 | 0 |
| R2/R4/NeSL | 5995 | 3.17 | 0.06 |
| RTE/Bov-B | 14555 | 7.79 | 0.15 |
| L1/CIN4 | 41462 | 28.60 | 0.55 |
| LTR elements: | 956994 | 1493.84 | 28.8 |
| BEL/Pao | 13627 | 13.23 | 0.26 |
| Ty1/Copia | 23572 | 19.55 | 0.38 |
| Gypsy/DIRS1 | 810313 | 1400.44 | 27.00 |
| Retroviral | 46095 | 34.45 | 0.66 |
| *DNA transposons* | 1674522 | 948.39 | 18.29 |
| hobo-Activator | 393570 | 266.43 | 5.14 |
| Tc1-IS630-Pogo | 786977 | 391.25 | 7.54 |
| En-Spm | 0 | 0 | 0 |
| MuDR-IS905 | 0 | 0 | 0 |
| PiggyBac | 11892 | 9.99 | 0.19 |
| Tourist/Harbinger | 53221 | 25.45 | 0.49 |
| Other (Mirage, P-element, Transib) | 0 | 0 | 0 |
| *Rolling-circles* | 5760 | 1.58 | 0.03 |
| Unclassified: | 3976735 | 1138.30 | 21.95 |
| Total interspersed repeats: |  | 4195.00 | 80.88 |
| Small RNA: | 0 | 0 | 0 |
| Satellites: | 10852 | 7.57 | 0.15 |
| Simple repeats: | 768 | 0.03 | 0 |
| Low complexity: | 0 | 0 | 0 |


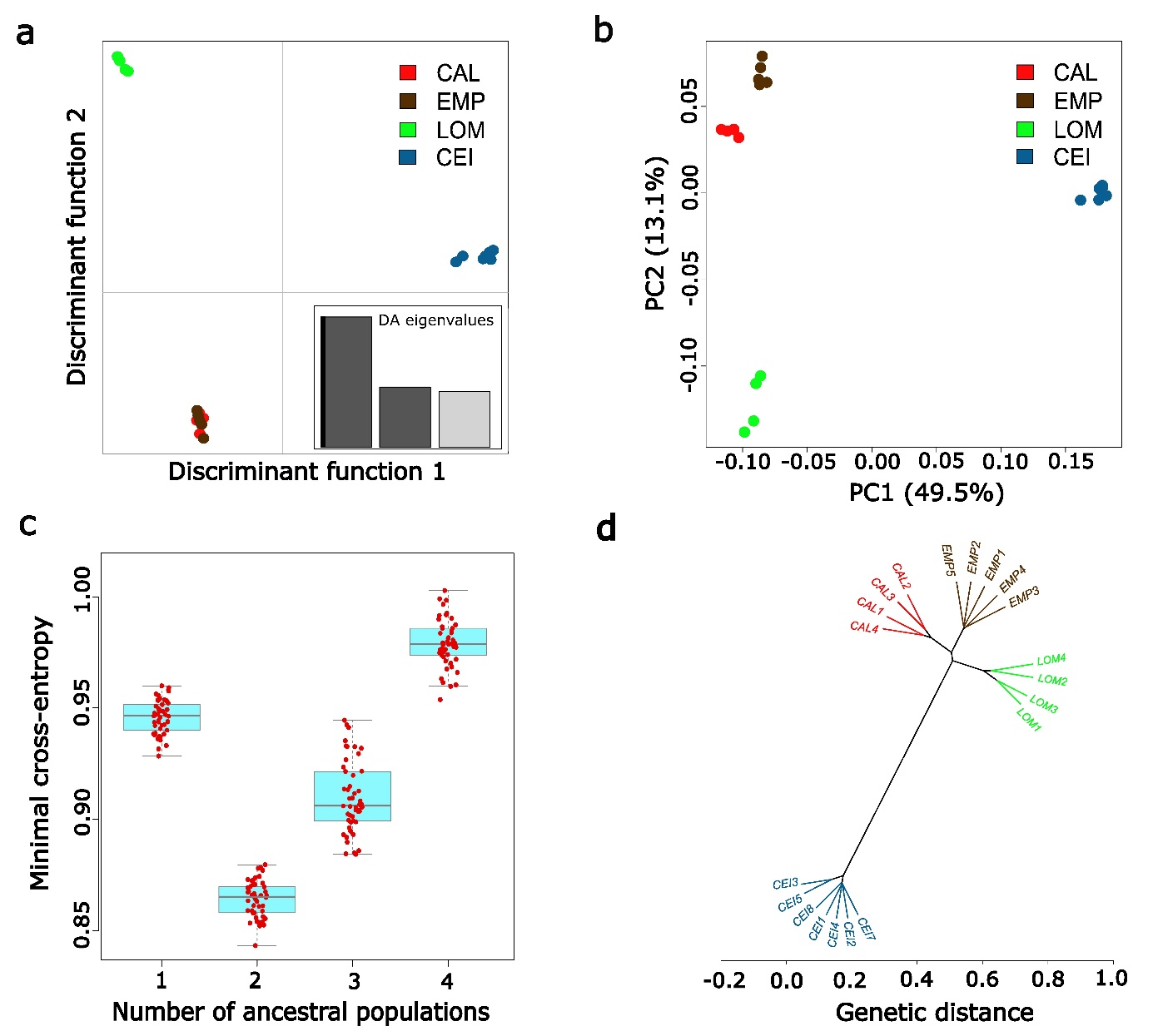


**Figure S2.** Population genetic structure of *Oophaga vicentei* in Panama estimated using a dataset of putatively 26 296 neutral SNPs*.* A) Discriminant Analysis of Principal Components. B) Principal Coordinates Analysis (PCoA) based on Nei´s genetic distance. C) Most likely number of ancestral populations calculated using the function *LEAce* from *sambaR* package, with 50 iterations. D) Unrooted Neighbor Joining tree based on a distance matrix of nucleotide diversity obtained using the function *findstructure* from *sambaR* package.


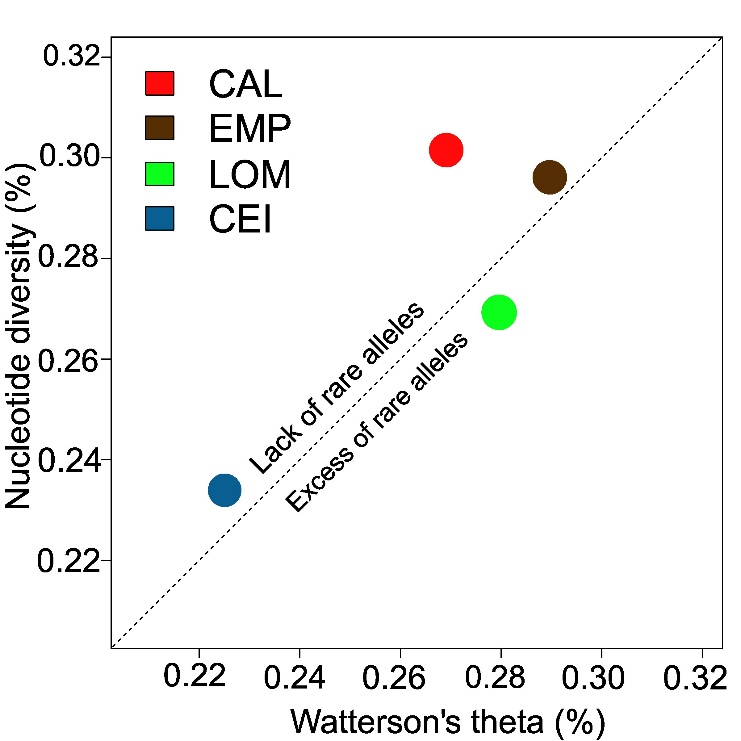


**Figure S3.** Population genetic diversity of *Oophaga vicentei.* Nucleotide diversity *vs* Watterson theta estimator of population mutation rate.

**References**

1. Velázquez-Zambrano CA. Análisis cariotípico de cuatro poblaciones de *Oophaga sylvatica* (Anura: Dendrobatidae). *Bachelor thesis.* Ecuador: Pontificia Universidad Católica del Ecuador; 2012.
